# The Prospective Lynch Syndrome Database reports enable evidence-based personal precision health care

**DOI:** 10.1186/s13053-020-0138-0

**Published:** 2020-03-14

**Authors:** Pål Møller

**Affiliations:** Department of Tumour Biology, The Norwegian Radium Hospital, Part of Oslo University Hospital, Oslo, Norway

**Keywords:** Inherited cancer, *MLH1*, *MSH2*, *MSH6*, *PMS2*, Prospective study, Lynch syndrome, Penetrance, Expressivity, Cumulative incidence, Gender, Personalized medicine, Precision medicine, Evidence based medicine

## Abstract

The aims of the Prospective Lynch Syndrome Database (PLSD) are to provide empirical prospectively observed data on the incidences of cancer in different organs, survival following cancer and the effects of interventions in carriers of pathogenic variants of the mismatch repair genes (*path_MMR*) categorized by age, gene and gender. Although PLSD is assumption-free, as with any study the ascertainment procedures used to identify the study cohort will introduce selection biases which have to be declared and considered in detail in order to provide robust and valid results. This paper provides a commentary on the methods used and considers how results from the PLSD reports should be interpreted. A number of the results from PLSD were novel and some in conflict with previous assumptions. Notably, colonoscopic surveillance did not prevent colo-rectal cancer, survival after colo-rectal, endometrial and ovarian cancer was good, no survival gain was observed with more frequent colonoscopy, new causes of cancer-related death were observed in survivors of first cancers due to later cancers in other organs, variants in the different *MMR* genes caused distinct multi-cancer syndromes characterized by different penetrance and phenotypes. The www.PLSD.eu website together with the InSiGHT database website (https://www.insight-group.org/variants/databases/) now facilitate evidence-based personalized precision health care for individual carriers at increased risk of cancer. The arguments are summarized in a final discussion on how to conceptualize current knowledge for the different practical purposes of treating cancers, genetic counselling and prevention, and for understanding /research on carcinogenetic mechanisms.

## Background

In 1985 it was suggested that inherited colon cancer should be termed Lynch Syndrome I, and inherited colon with extracolonic cancers Lynch Syndrome II [1]. (OMIM **#** 120435). In 1989 an international network of researchers (ICG-HNPCC) set out to identify the genetic variants causing what they termed the Hereditary Non-Polyposis Colon Cancer (HNPCC) syndromes [2]. It was discovered that a major fraction of HNPCC tumours were characterised by micro-satellite instability (MSI) and caused by inherited pathogenic variants (path_) affecting the mismatch repair (*MMR*) genes. In 2009 the term Lynch Syndrome (LS) was redefined to denote this hereditary condition [3]. That paper, however, erroneously stated that LS was identical to HNPCC, while in fact variants in several non-*MMR* genes cause HNPCC without MSI tumours. In 2009 another group stated that Lynch syndrome includes both individuals with an existing cancer and those who have not yet developed cancer [4]. These different definitions have created conceptual confusion, especially the latter because Mendelian inheritance by definition is describing inherited traits (phenotypes). How to explain the original nomenclature to integrate the concept of probability by age to demonstrate an inherited trait is challenging and may be why the discussions on inherited cancers have separated from the networks for inherited disorders diagnosable at birth or in infancy. Nomeclature for LS should comply with consented medical concepts delineating diseases from normal variation, and nomenclature should be applied as for the other inherited cancer and inherited disease syndromes. Using the same annotation for healthy carriers as for cancer cases is confusing and may be misunderstood and in conflict with both the scientific, ethical and legal platforms of medical genetics. Without defined and consented concepts and nomenclature communication to reach consensus is difficult.

ICG-HNPCC established the Amsterdam I clinical criteria to identify families with highly penetrant and dominantly inherited colon cancer. *Path_MLH1* and *path_MSH2* variants were identified as causative in some such families. Based on the logical circle that returned the selection criteria as results, it was concluded that LS was a dominantly inherited colorectal cancer (CRC) syndrome with high penetrance. It became clear that endometrial cancer was part of LS [5] and the revised Amsterdam II clinical criteria were agreed, including endometrial cancer as an affected phenotype [6] and consistent with *path_MSH6* being a cause of LS. It soon became evident, however, that the Amsterdam criteria were insensitive in identifying LS families caused by *path_MLH1* or *path_MSH2* variants, and even less sensitive in identifying LS caused by *path_MSH6* or *path_PMS2* variants [7]. Despite these shortcomings, these clinical criteria are still in use as a clinical pre-test to select cases for genetic testing. The result has been that most LS families identified historically have fulfilled these criteria and have dominantly inherited CRC/endometrial cancer with high penetrance, while relatively few *path_MSH6* and very few *path_PMS2* families have been identified. It also became clear that while in former generations most patients died from their first cancers, a substantial number now survive their first cancer and live on to develop further cancers that are often in other organs. In summary, knowledge of LS a decade ago was by and large derived from retrospective family studies based on questionable concepts as were the clinical guidelines on how to manage both healthy *path_MMR* carriers and affected LS patients [8]. Because it was recognized that colonoscopy conducted every 3 years did not fully prevent CRC, guidelines were revised advocating a reduction of the interval between colonoscopies to 1–2 years, with no evidence that this would reduce CRC incidence.

Researchers from several collaborating European centres agreed to establish the PLSD during a meeting in Palma, Mallorca on May 4th 2012. The aims were to challenge and test assumptions based upon retrospective information, to determine empirical prospectively observed cancer incidences and survival in *path_MMR* carriers and to observe the effects of interventions and categorize these by age, gene and gender.

## Methods

To validate the assumptions upon which clinical guidelines were based, the data entered into PLSD had to be assumption-free. The data recorded included gender, age of inclusion, age last observation, age at death, diagnosis of any cancer, age at diagnosis of cancer and the inherited *path_MMR* variant that had been identified. The data had to be complete for these variables, and all carriers known at each reporting centre had to be contributed. Later, cancer stage at diagnosis and time since last colonoscopy at cancer diagnosis were requested for all prospectively detected CRCs and added to the information already filed. Reported pathogenic variants were assumed germline. The data were included in an Oracle relational database. Details relevant to an understanding of its capabilities and interpretation of outputs are discussed in our previous reports [9, 10].

To control *lead-time bias*, all cancers diagnosed at the same age as inclusion were considered prevalent (first round cancers), and all cancers diagnosed later were scored as prospective. Some carriers had been followed for a long time, and there are *time-trend biases* in the technical development of the screening techniques that were applied, in understanding of what to look for during screening and in changing intervals between colonoscopies. There are *length-time* biases when no obligatory examinations were undertaken at right-censoring observation time. Length-time bias will most probably result in an artificially low incidence of CRC. The longer the observation time, the more impact time-trend biases will have, and the less impact lead- and length-time biases will have. Generally, in screening trials, there should be a randomized control group, but this approach is considered impossible for ethical reasons in LS carriers. Time-trend and length-time biases were accepted in order to maximize the number of observation years. Updated information on the carriers filed in the PLSD may be added to re-analyse the series, correcting for time-trends and length-time bias.

Survival was measured as overall/crude survival, because disease-specific survival includes assumptions.

Any study has a selection procedure to identify the cohort to be studied – *a selection bias*. Results from any study should be interpreted based on the selection procedures, to avoid returning the selection criteria as the results of the studies. A selection artefact included in the PLSD dataset is that genetic testing was usually done in cancer families: there may be additional genetic and/or environmental factors causing disease in such families [11] resulting in artificially high prospective average cancer incidences in carriers. A selection bias is the low number of low-penetrant variants. This bias may also be considered a result demonstrating the low penetrance of these variants.

Based on power calculations, the first PLSD dataset was censored when 25,000 observation years had been filed, and the first three descriptive papers were published: 1) incidence rates for cancers in carriers without prior or prevalent cancers [12], 2) incidence rates for cancers in carriers who had prior and/or prevalent cancers [13], and 3) - because papers 1 and 2 gave similar results – a combination of the first two papers into one study including all carriers with or without cancer prior to or at inclusion [14]. With these three papers the original goal was reached. When an additional independent series of about 25,000 observation years were filed, we compared this independent replication cohort with the first series, reaching the conclusion that the results were similar. We then combined all cases in one large data set, refining our estimates of cancer risk and survival by age, gene and gender [15]. At that time more contributors expressed their interest in participating, and the PLSD database is still growing.

In addition to the four descriptive reports described above, three hypothesis-testing papers have been published: CRC incidence related to the interval between colonoscopies [16], clinic-pathological stage of colon cancer related to time since last colonoscopy [17] and survival after colon cancer related to time since last colonoscopy [18].

## Some results [12–18] of interest

### Colonsocpy with removal of adenomas did not apparently reduce colorectal cancer incidence

As detailed in Table 1, the prospectively observed incidences of colorectal cancer demonstrated by PLSD was not different from retrospective studies as contrast groups [19–21]. These retrospective studies were based on three generations but without notion on carriers included possibly having been subjected to colonoscopy. Assuming 7 years follow-up time for the last generation in the families reported corresponding with the average follow-up time reported to PLSD and substituting these with the average incidences reported by PLSD would, however, probably not change their reported results. The PLSD results are in conflict with the belief that colonoscopy compliant with the world-wide advocated clinical guidelines prevent CRC in the carriers. It is a challenge to clarify why this is so.

**Table 1:** Cumulative risk at 70 years for colo-rectal cancer (CRC), endometrial cancer and ovarian cancer in three retrospective studies of carriers [19–21] and prospective findings in carriers followed-up by colonoscopy reported by PLSD [15]

Cancer	Study	Gender	70 years cumulative incidence (95% confidence interval)			
			*Path_MLH1*	*Path_MSH2*	*Path_MSH6*	*Path_PMS2*
CRC	Bonadona et al. [19]	both genders	41% (25–70%)	48% (30–77%)	12% (8–22%)	
	Dowty et al. [20]	males	34% (25–50%)	47% (36–60%)		
		females	36% (25–51%)	37% (27–50%)		
	Ten Broeke et al. [21]	males				13% (8–22%)^a^
		females				12% (7–21%)^a^
	PLSD [15]	males	53% (45–62%)	46% (37–59%)	12% (5–35%)	3% (1–35%)
		females	44% (37–52%)	42% (35–50%)	20% (12–41%)	
Endometrial cancer	Bonadona et al. [19]	females	54% (20–80%)	21% (8–77%)	16% (8–32%)	
	Dowty et al. [20]	females	18% (9–34%)	30% (18–45%)		
	Ten Broeke et al. [21]	females				13% (7–24%)^a^
	PLSD [15]	females	35% (29–43%)	47% (38–56%)	41% (29–58%)	13% (5–50%)
Ovarian cancer	Bonadona et al. [19]	females	20% (1–65%)	24% (3–52%)	1% (0–3%)	
	Dowty et al. [20]	females	13% (6–26%)	10% (4–21%)		
	Ten Broeke et al. [21]	females				Not increased
	PLSD [15]	females	11% (7–17%)	17% (12–27%)	11% (4–33%)	3% (1–43%)

^a^Cumulative risk at 80 years

### Early diagnosis and treatment cured most colorectal cancer cases

The goal – in conflict with the goal for breast cancer screening in *path_BRCA1/2* carriers – has been to prevent CRC, not to cure. Colonscopy with adenomectomy every 3 years or more often, would have been a success story if the goal had been to cure CRC. But we as experts had promised ourselves, the carriers and those paying for health care that colonoscopy would prevent, not cure, CRC.

### Colonsocopy repeated more frequently than every 3 years neither reduced colorectal cancer incidence, nor stage of colorectal cancer at diagnosis, and did not improve survival

Because the proposed accelerated adenoma-carcinoma pathway in LS was supported by a previous prospective study [22], a reduced CRC incidence was expected in patients receiving more frequent colonoscopy. The lack of such a reduction in incidence suggests that another mechanism with the opposite effect may be operating: overdiagnosis. Biological mechanisms that would make this mechanism possible have been demonstrated recently: LS carriers have multiple MMR deficient crypts in macroscopically normal gut surface, only some of which eventually develop into cancer and may do so without a macroscopically visible non-invasive precursor [23]. Both the MMR deficient crypts and cancers are targeted by the host immune system, and modern immunotherapy may shift the balance between the tumour and the host immune system to fight established MSI cancers. In summary, the PLSD epidemiological observations indicate that LS-associated tumours may disappear, and there is growing evidence for biological mechanisms that may mediate this.

#### Incidence of endometrial cancer is high and prognosis is good

This means that although in former generations most female carriers died from either CRC or endometrial cancer, they now usually live on and develop cancers in other organs.

#### Competitive causes of death

Current outcomes for survivors of CRC and endometrial cancers cannot be obtained from retrospective studies because of the low number of survivors in previous generations. This is probably why, in previous retrospective studies the high incidence of urothelial cancers in *path_MSH2* carriers was not clearly described, the lower incidence of CRC in female than male *path_MSH2* carriers probably was an artifact due to competing causes of death, and the later onset prostate cancers were also missed because of competing causes of death.

#### *Path_MSH6 *variants cause a sex-limited dominantly inherited cancer syndrome

In *path_MSH6* carriers the cumulative risk for endometrial cancer is high, while the risk for CRC is much lower both in men and women. In summary, the cancer incidence is high in females and much lower in males. In *path_MSH6* kindreds most males are unaffected resulting in clinically ‘skipped generations’, and families were not identified by clinical criteria [7]. As a consequence, when genetic testing was restricted to those meeting the clinical criteria, *path_MSH6* families were usually not identified.

#### Breast cancer incidence is slightly and equally increased in all carriers

This is as expected if *path_MMR* variants do not cause breast cancer but carriers are subject to over-diagnosis by mammographic screening.

#### *Path_PMS2* variants do not cause LS

The incidence of cancer is so low in *path_PMS2* carriers, that according to the definition of LS as a dominantly inherited cancer syndrome with high penetrance [3, 24], *path_PMS2* variants do not cause LS. *Path_PMS2* variants are the major cause for the recessively inherited CMMRD syndrome presenting in adolescence [25] and a slightly increased incidence of related phenotypes in heterozygous carriers of recessively inherited diseases (heterozygote manifestations) is no novelty.

#### Low penetrance pathogenic *MMR* variants

The InSiGHT criteria for identifying pathogenic *MMR* variants are tailored to identify high penetrance variants causing dominantly inherited disorders: low penetrance variants may be more frequent than 1% and will by the consented criteria be classified as normal variation [26]. Such may, however, cause recessively inherited disorders – cfr. discussion above on *path_PMS2.* We have no criteria for identifying low-penetrance variants, no criteria to separate them from normal variation, no criteria to distinguish low penetrance pathogenic variants from those of high penetrance, and correspondingly we have no nomenclature to denote low-penetrance variants. In consequence we do not know how frequent *path_PMS2* variants are because we do not know how to identify them. The retrospective studies in *path_PMS2* carriers demonstrated in CRC kindreds demonstrate CRC incidence comparable with what is observed in CRC kindreds without demonstrable genetic cause(s) [21]. There is a low risk for endometrial cancer [12–15, 21] and *path_PMS2* carriers for a founder variant have an increased risk for late onset CRC [27].

#### Ovarian cancer in LS has good prognosis

Three out of four ovarian cancers in LS were cured. The incidence in *path_MSH6* carriers is low and not measurable in *path_PMS2* carriers. These observations question the clinical advice to undertake prophylactic oophorectomy which was based on assuming the same mortality as in *path_BRCA1/2* associated ovarian cancer [28–30]. An analysis of prophylactic hysterectomy and oophorectomy reported to the PLSD and current clinical guidelines for risk-reducing surgery in the collaborating centres are currently in progress.

#### Urinary tract and prostate cancers

Ureter and urinary bladder cancers are frequent especially in *path_MSH2* carriers, and male *path_MSH2* carriers have an additional approximately 25% lifetime risk for prostate cancer. Emerging evidence indicates that carriers of pathogenic variants of many other DNA-damage repair genes are also at risk for urothelial cancers [31].

#### Causes of death in LS have changed

Table 2 indicates the probabilities for LS carriers of dying from cancers affecting different organs, calculated from the incidence of cancer in each organ multiplied by the observed 10-years incidence of dying from each cancer. In contrast to the situation in former generations where most carriers died from their first cancer in the colon or endometrium, the overwhelming majority of prospectively diagnosed patients within follow-up programs now survive their first cancers. They live on to develop new cancers in other organs. This new information cannot be obtained from retrospective studies of former generations. These cancers are, to a large degree, gene-specific and some have a serious prognosis. Upper-gastro-intestinal cancers (gastric, duodenum, bile duct and pancreas) are emerging as significant causes of death in *path_MLH1* carriers, while urinary tract and brain tumours emerge as causes of death in *path_MSH2* carriers. The figures in Table 2 are derived from the report from first PLSD series specifying cancer in each organ [14], more detailed risks for the later onset extracolonic cancers will be specified in upcoming PLSD reports. *Path_MSH6* and *path_PMS2* carriers have risks that are so low that when cured from CRC or endometrial cancers, any increased risk for other cancers is hardly measurable.

**Table 2:** Risk of dying from cancer in each organ before 80 years of age calculated as cumulative risk 70 years multiplied by [1-(10 years survival)] [14], both genders combined. *Path_PMS2* carriers not included because too few prospective cancers before 70 years of age for meaningful calculations

ICD9	Organ	Cumulative incidence by age 70 years			10 years survival	Risk of dying from before 80 years (cumulative incidence 70 years)∙[1-(survival)]		
		path_MLH1	path_MSH2	path_MSH6		path_MLH1	path_MSH2	path_MSH6
153	Colon	42%	40%	14%	88%	5%	5%	2%
154	Sigmoid and rectum	9%	14%	5%	70%	3%	4%	2%
182	Endometrium	40%	53%	46%	93%	3%	4%	3%
183	Ovaries	10%	17%	13%	74%	3%	4%	3%
151	Stomach	6%	4%	1%	61%	2%	2%	0
152	Duodenum	4%	2%	0	67%	1%	1%	0
156	Bile duct and gall bladder	4%	0	0	14%	3%	0	0
157	Pancreas	4%	1%	1%	0	4%	1%	1%
188	Urinary bladder	4%	6%	4%	81%	1%	1%	1%
189	Ureter and kidney	4%	16%	3%	71%	1%	5%	1%
174	Breast	12%	12%	13%	89%	1%	1%	1%
185	Prostate	13%	13%	4%	80%	3%	3%	1%
191	Brain	1%	2%	1%	22%	1%	2%	1%

#### www.PLSD.eu enables individualized evidence-based precision medicine

The method used to calculate probabilities for cancer from 25 years of age onwards in the published reports may be used to calculate risk from any given age onwards. The InSiGHT variant database (http://insight-database.org/) indicates which variants in the genes are pathogenic, while the www.plsd.eu website interactively enables the user to obtain probabilities for cancer in any organ by indicating an individual’s age, gene and gender. Together the databases enables evidence based individualized precision medicine for the carriers. The PLSD website is embedded in the InSiGHT variant database website and can be launched by selecting the tab ‘MMR CANCER RISK’.

## What is Lynch syndrome?

The definition of LS has changed repeatedly. Currently used definitions are contradictory, in conflict with ethical and scientific paradigms, and some results provided by PLSD are in conflict with all of them. The definition ‘*Lynch syndrome is a highly penetrant hereditary cancer syndrome caused by pathogenic germline variants in DNA mismatch repair (MMR) genes’* [24] excludes male *path_MSH6* carriers and all *path_PMS2* carriers. Because of their biological similarities and responses to treatment, one may suggest to consider all MSI CRC cases as LS, if so most cases would not be inherited. If considering families with clinically dominantly inherited MSI tumours as LS, not all families have demonstrable pathogenic *MMR* variants [32]. Variants in additional DNA repair genes cause urothelial cancer [31]. Also, it is increasingly evident that different classes of variants in the *MMR* genes are associated with different penetrance – the emerging evidence for variants associated with differential splicing being one example [33] and which may be more frequent than is currently recognized [34]. Gene panel testing in both blood and tumours will identify many variants in these genes in incident cancer cases and there is a need to conceptualize and categorize interpretation of the results. The umbrella term ‘Lynch syndrome’ has been practically and scientifically useful but may longer be so. It appears timely to reconsider data from all sources in relation to LS and to be more precise in how we define it. For example, it may be clinically practical to group cancer cases who will benefit from similar treatment modalities. Better defined and individualized prospective probabilities of cancer may be needed for genetic counselling and planning of preventive interventions. Understanding associations between genetic variants and carcinogenetic and biological mechanisms may be objectives for further research. These topics are overlapping but not identical and will have different outputs relevant to decision-making in these different contexts.

## Data Availability

No new data or material.
